# Institutional Housekeeping

**Published:** 1911-11-04

**Authors:** 


### INSTITUTIONAL HOUSEKEEPING.

(Contributions invited: Workers' Questions will be welcome.)

### I.—The Art of Buying.

GOOD TEA, AND HOW TO RECOGNISE IT.

Of late years the standard of tea used in institutions has been considerably raised, but still in very many there is much room for improvement. One main cause of failure lies in the attempt to buy tea at an impossibly low price. We have known as little as 9½d. per lb. to be paid, though it is fair to observe that 11½d. per lb. was the lowest price paid in the 106 hospitals dealt with in the Report of the King's Fund. And when a better price is paid, say 1s. 2d. per lb. for a chest of about 100 lb., it is often supplied by a local small grocer without experience, who aims at making an excessive profit.

The majority of housekeepers have no knowledge by what signs a sample of tea should be chosen.

Tea may be judged by the organs of sight, smell and taste. The dry leaves should be well twisted, crisp to the touch, and free from much stalk, for stalk floats on the surface, and imparts nc flavour. They should also be free from dust. An expert would be able to tell by smelling the dry leaves whether in the manufacture they have been over-fermented or burnt by over-firing, and this faculty can be learned by noting the scent of really good tea and comparing it with that of inferior qualities. Having carefully observed the dry leaves, an infusion must be made, and care taken to notice whether the tea leaf yields a dull, dark, flat liquor; whether there is much colour, but no taste; whether the liquor is blackish; whether the tea-leaves after infusion turn very dark. Any of these signs denote tea of poor quality. Good tea should yield liquor of a reddish gold tint, full-bodied, yet not muddy; it should turn a rich chocolate colour when milk is added, and the used leaves should have a bright brown, not black, shade.

Every housekeeper should be on the watch for indications of adulteration in low-priced teas. The leaves are sometimes moistened, warmed, and powdered, colouring matter, such as indigo, Prussian blue, turmeric, etc., being then shaken over and mixed through them. This is done to improve the colour, and may be detected by shaking the leaves in cold water and quickly draining it off into another vessel. The sediment can then be examined, and subjected, if desired, to a laboratory test. Used tea leaves are also often added to give bulk; they can be discovered by analysis.

Tea quickly deteriorates by exposure to air and by being kept near strong-smelling goods, such as soap, cheese, oranges, onions, etc. It should be stored in air-tight tins or glass bottles in a perfectly dry, fairly warm place.

Sometimes through carelessness or accident the leaves acquire some objectionable flavour. To remove this the tea must be spread out on trays or paper, and remain exposed to the air for thirty-six hours. Should tea become damp, and lose its crispness, not an uncommon occurrence at the sea or when carelessly stored, the leaves should be spread thinly on metal trays, and set on the plate rack for four or five hours.

It is important in selecting tea to bear in mind the nature of the water with which it will be made. Teas that suit soft water will usually prove a failure in hard water, and the housekeeper must make sure that all the goodness can be extracted in the water available for use so as to avoid waste of time and money. Strong Assams are usually considered best for hard water, and Ceylons and Darjeelings are suitable for soft water.

### The Importance of Counting Heads.

The futility of presenting housekeeping totals without reckoning numbers is becoming more apparent every year, and the statistics collected by the King's Fund are doing much to demonstrate this to institutions. We hear a good deal of the reduction in housekeeping expenses, and more especially in the price of food during the last few years. We are not told how much of this is due to the fact that under the new system the board of all laundrymaids is deducted from that of the household and placed to the laundry account. Nothing would be easier than to dodge the critic in such matters, if it were desired to show a decrease in provisions alone, for there are workers in every hospital who can either be boarded very much to their own comfort in the institution or allowed to find their own meals.

The only road to a thorough grasp over the commissariat lies in comparing the expenses of one quarter with another, better still one week with another, and as soon as an attempt is made to do this the question of numbers leaps into paramount importance. For instance, in one hospital, the spring quarter, January to March 1911, shows an increase in cost of provisions over the corresponding quarter last year of £30. In an institution where heads are not accurately numbered the authorities will be told the staff was increased by several extra nurses during this period and will leave it at that. But in a household where knowledge is preferred to guesswork, the housekeeper will state that five extra nurses were boarded during a period of ten weeks, accounting for £17 10s. of the surplus cost. She still has an increase of £12 10s. to account for, and must compare the items of one quarter with those of the corresponding one until she discovers how it has come to pass that she has been spending this extra £1 a week. To discover this is half way to saving it, unless the extra cost is entailed by a very small but steady increase in prices over which she has no control.

